# Production of patient‐specific electron beam aperture cut‐outs using a low‐cost, multi‐purpose 3D printer

**DOI:** 10.1002/acm2.12421

**Published:** 2018-07-26

**Authors:** Steven Michiels, Bram Mangelschots, Robin De Roover, Cédric Devroye, Tom Depuydt

**Affiliations:** ^1^ Laboratory of Experimental Radiotherapy Department of Oncology KU Leuven – University of Leuven Leuven Belgium; ^2^ Department of Radiation Oncology University Hospitals Leuven Leuven Belgium

**Keywords:** 3D printing, aperture cut‐out, collimation, electron beam radiotherapy

## Abstract

Electron beam collimators for non‐standard field sizes and shapes are typically fabricated using Styrofoam molds to cast the aperture cut‐out. These molds are often produced using a dedicated foam cutter, which may be expensive and only serves a single purpose. An increasing number of radiotherapy departments, however, has a 3D printer on‐site, to create a wide range of custom‐made treatment auxiliaries, such as bolus and dosimetry phantoms. The 3D printer can also be used to produce patient‐specific aperture cut‐outs, as elaborated in this note. Open‐source programming language was used to automatically generate the mold's shape in a generic digital file format readable by 3D printer software. The geometric mold model has the patient's identification number integrated and is to be mounted on a uniquely fitting, reusable positioning device, which can be 3D printed as well. This assembly likewise fits uniquely onto the applicator tray, ensuring correct and error‐free alignment of the mold during casting of the aperture. For dosimetric verification, two aperture cut‐outs were cast, one using a conventionally cut Styrofoam mold and one using a 3D printed mold. Using these cut‐outs, the clinical plan was delivered onto a phantom, for which the transversal dose distributions were measured at 2 cm depth using radiochromic film and compared using gamma‐index analysis. An agreement score of 99.9% between the measured 2D dose distributions was found in the (10%–80%) dose region, using 1% (local) dose‐difference and 1.0 mm distance‐to‐agreement acceptance criteria. The workflow using 3D printing has been clinically implemented and is in routine use at the author's institute for all patient‐specific electron beam aperture cut‐outs. It allows for a standardized, cost‐effective, and operator‐friendly workflow without the need for dedicated equipment. In addition, it offers possibilities to increase safety and quality of the process including patient identification and methods for accurate mold alignment.

## 1 INTRODUCTION

For a long time, electron beam therapy has been an important, cost‐friendly modality for the irradiation of superficial tumors, with minimal dose to the underlying normal tissues.^1^, ^2^ Modulation of the electron fields may improve the dose conformity 3 and has been extensively investigated using the photon multi‐leaf collimator (MLC) 4, 5, 6 or a dedicated electron MLC.^7^, ^8^ Nevertheless, the use of static electron fields remains common 9 and collimation is typically performed using aperture cut‐outs placed in the electron applicator at a small distance from the patient's skin.^10^

Collimators for non‐standard field sizes and irregular shapes are typically cast in Cerrobend using patient‐specific Styrofoam molds. These molds are often produced using a dedicated foam cutting machine, which may be expensive and mainly serves a single purpose. In addition, Styrofoam panels are bulky and require dedicated storage space, which represents a cost as well. Additive manufacturing or 3D printing may offer a valuable alternative solution for these conventional, machine cut Styrofoam molds.

An increasing number of radiation oncology centers have a 3D printer available on‐site, for instance to create individualized bolus,^11^ dosimetry phantoms^12^ and immobilization.^13^ 3D printing likewise allows to produce the molds for the production of patient‐specific aperture cut‐outs. As such, a single, relatively inexpensive device with a single raw material type can be used to create a wide range of patient‐specific treatment auxiliaries. This proof of concept study describes a 3D printing solution for the production of patient‐specific aperture cut‐outs, including a dosimetric comparison with conventionally produced apertures.

## 2 MATERIALS AND METHODS

### 2.A Clinical plan

**Figure 1:**
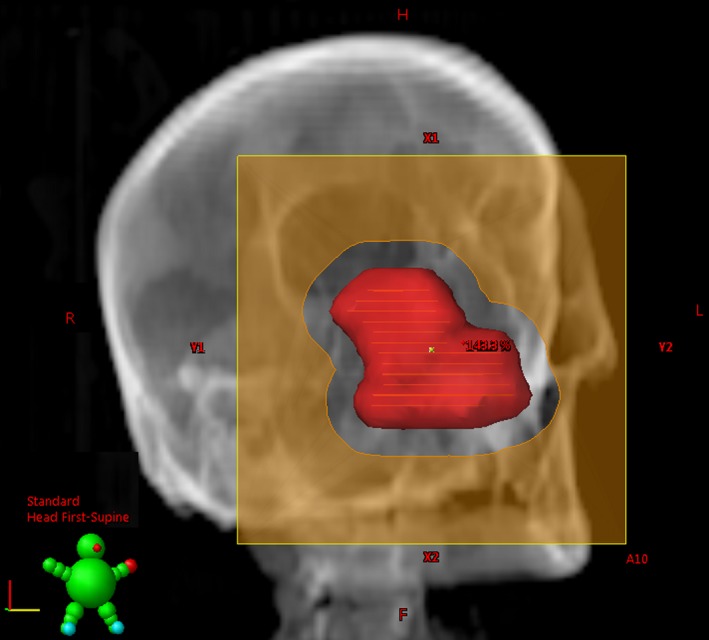
Beam's eye view of the planning target volume and the aperture for the described case.

The data of a patient with recurring squamous cell carcinoma of the nasal cavity, treated with 33 fractions of 2 Gy, were used retrospectively. The used treatment machine was a Clinac 2100C/D (Varian Medical Systems, Palo Alto, CA, USA). The clinical plan in the Eclipse (Varian) treatment planning system (TPS) consisted of a single 12 MeV electron beam, perpendicular to the body surface and with a source‐to‐surface distance of 110 cm. The gantry and collimator angle were 340° and 270°, respectively. The aperture was designed conformal to the beam's eye view of a 7 mm isotropic expansion of the planning target volume, fitting in a 10 × 10 cm² applicator tray (see Fig. 1).

### 2.B 3D printing workflow for aperture cut‐out production

**Figure 2:**
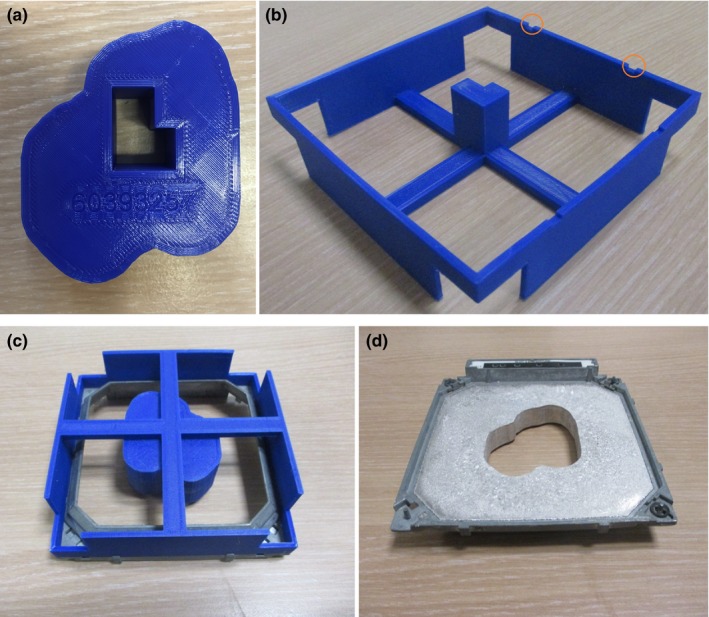
(a) 3D printed mold with integrated patient ID‐number and key for uniquely fitting to a positioning device. (b) This latter is 3D printed as well, is reusable and contains a number of grooves for uniquely fitting to the applicator tray. (c) This consequently assures correct and error‐free alignment of the combined assembly during casting of (d) the aperture cut‐out.

The RT plan DICOM‐file was exported from the TPS. An in‐house developed script in the open‐source software Python(x,y) (version 2.7.10, SciPy.org) extracted and processed the required information for each treatment field from the DICOM: the aperture contour points projected on the isocentre plane, the collimator angle, the patient identification number, the source‐to‐isocentre and the source‐to‐tray distance. Using the FreeCAD open‐source add‐on, the script backprojected the contour points to the applicator tray plane and created a mold in .STL‐format by extruding the contour 25 mm along the beam axis. The patient ID‐number was integrated in the mold and an asymmetric key was foreseen to attach the mold uniquely fitting to a positioning device [Fig. 2(a)]. The positioning device, reusable and 3D printed as well, fitted uniquely onto the applicator by means of a number of grooves [Fig 2(b)]. As such, correct and error‐free alignment of the combined assembly was assured [Fig. 2(c)] during casting of the aperture cut‐out [Fig. 2(d)].

### 2.C 3D printer settings

The .STL‐file of the mold was converted to GCODE, printable on a fused depositioning modeling 3D printer (type N2 Plus, Raise3D, Costa Mesa, CA, USA), using the ideaMaker slicing engine software (version 3.1.6, Raise3D). The main slicer settings were an infill density of 5%, four shells and a layer height of 0.25 mm. A raft was added by the software to enhance adhesion of the 3D printed mold to the printer bed. The commonly used polylactic acid (PLA) was used as filament material for the 3D printer, using an extrusion temperature of 215°C and a heated bed temperature of 60°C. The melting point T_m_ of PLA amounts to around 150°C, allowing it to withstand the hot Cerrobend (T_m_ ≃ 70°C) during casting of the aperture cut‐out.

### 2.D Dosimetric validation

Two aperture cut‐outs were available for the clinical plan: one using the 3D printed mold and positioning device and one using the conventional clinical workflow with a Styrofoam mold. Using these aperture cut‐outs, the clinical plan was delivered onto a polystyrene slab phantom (RW3, PTW, Freiburg, Germany) using twice the amount of monitor units, with radiochromic film (type Gafchromic EBT3, Ashland Advanced Materials, Bridgewater, NJ, USA) positioned perpendicular to the beam axis at a clinically relevant depth of 2 cm. Calibration and processing of the films was performed as described elsewhere.^14^ The gamma‐index agreement score 15, 16 between both delivered fields was calculated using 1.0% (local) dose‐difference and 1.0 mm distance‐to‐agreement acceptance criteria.

## 3 RESULTS

**Figure 3:**
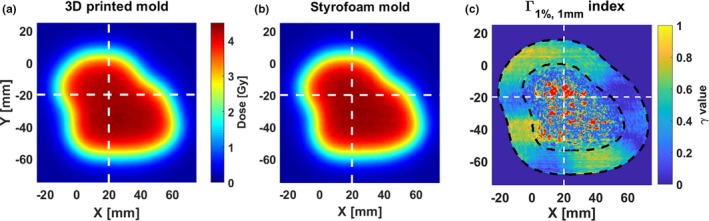
(a) Acquired electron beam dose distribution at 2 cm depth using the aperture cut‐out produced with the 3D printed mold & positioning device and (b) the conventional Styrofoam mold, respectively. The white‐dashed lines mark the position of the line profiles shown in Fig. 4. (c) Gamma‐index values using 1% local dose‐difference and 1 mm distance‐to‐agreement acceptance criteria. The red pixels are the pixels with a gamma‐index ≥1. The black‐dashed lines are the boundaries of the (10%–80%) dose region, for which the gamma‐index agreement score was 99.9%.

**Figure 4:**
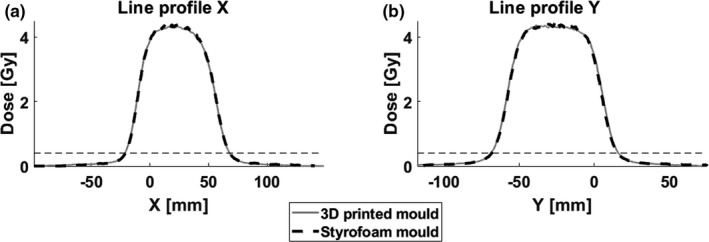
Line profiles along the (a) *X*‐ and (b) *Y*‐axis using the aperture cut‐outs produced with the 3D printing workflow and with the conventional workflow using Styrofoam.

Figures 3(a) and 3(b) show the acquired dose distribution at 2 cm depth using the aperture cut‐out once realized conventionally with a Styrofoam mold and once with a 3D printed mold and positioning device. Figure 3(c) shows the corresponding Γ_1%, 1 mm_—index distribution, for which an agreement score of 96.2% was found. The red pixels are the pixels with a gamma‐index ≥1, nearly all of which are located within the central high dose region. The failing of these pixels is attributed to the local dose differences due to residual noise in the film measurements.^14^ When taking into account only the pixels relevant for the current proof of concept study, that is, the (10%–80%) dose region marked by the black‐dashed lines in Fig. 3(c), the agreement score was 99.9%. The white‐dashed lines mark the position of the line profiles, which are shown in Figs. 4(a) and 4(b).

## 4 DISCUSSION

In this technical note, a proof of concept for the production of patient‐specific electron beam aperture cut‐outs using 3D printing technology has been elaborated. The proposed workflow allows for the production of aperture cut‐outs without dedicated foam cutting equipment and without dedicated foam material, both of which may represent a significant cost in terms of monetary or storage resources. Instead, a single, relatively inexpensive and versatile 3D printer with a single raw material can be used to create a wide range of patient‐specific treatment auxiliaries besides the molds for aperture cut‐out production, such as individualized bolus,^11^ dosimetry phantoms,^12^ and immobilization.^13^ In addition, the proposed workflow allows for introduction of a number of measures to enhance safety and quality in the aperture production process, including patient identification and accurate, error‐free alignment during casting.

A dosimetric verification was included in this study, comparing the dose distributions obtained with an aperture cut‐out cast using a 3D printed mold and a conventional Styrofoam mold, respectively. A gamma‐index agreement of 99.9% was found in the dose region relevant for this proof of concept study, that is, the penumbra region. A limitation of this study is that only one such direct dosimetric comparison between both workflows was performed. For the performed measurement, however, the agreement score in the penumbra region was excellent. In addition, the proposed 3D printing workflow produces a mere geometric reproduction of the mold's contour generated with the conventional workflow, for which no dosimetric differences should be expected.

Following this proof of concept study, the 3D printing workflow for the production of patient‐specific aperture cut‐outs has been clinically implemented and is now in routine use at the author's institute. Aperture cut‐outs for more than 80 clinical electron beam fields have been produced, for applicator tray sizes ranging from 6 × 6 cm² to 20 × 20 cm². The drawback of the 3D printing workflow is the longer time required to produce the 3D printed molds compared with the conventional Styrofoam molds. Using the described slicer settings, the 3D printing time ranges between 1 h and 9 h, depending on the size of the mold. At the authors’ institute, however, the convention is to have clinical treatment plans prepared 1 day before the first day of treatment. As the 3D printing process can continue overnight and as several molds can be printed in batch, this allows for timely completion of the 3D printed mold before the start of treatment.

A reduction in the 3D printing times may be achieved optimizing the slicing engine settings. More importantly, the proposed workflow may be further enhanced by scripting the creation of the STL‐file for the mold in the TPS. This is enabled in the authors’ institute's TPS using C# (Microsoft Corporation, Redmond, WA, USA) programming language and is currently being pursued. As such, a streamlined workflow can be envisioned in which the required mold can be exported straight from the TPS, converted to GCODE and sent directly to a 3D printer.

In summary, 3D printing allows for a standardized, operator‐friendly workflow for the production of patient‐specific electron beam aperture cut‐outs without the need for specific equipment to fabricate molds. The proposed workflow includes possibilities to increase safety and quality of the process including patient identification and methods for accurate mold alignment.

## ACKNOWLEDGMENT

S. Michiels was supported by a grant from Kom op tegen Kanker (Stand up to Cancer), the Flemish cancer society.

## CONFLICT OF INTEREST

The authors have no other relevant conflicts of interest to disclose.
